# Fingerprinting cities: differentiating subway microbiome functionality

**DOI:** 10.1186/s13062-019-0252-y

**Published:** 2019-10-30

**Authors:** Chengsheng Zhu, Maximilian Miller, Nick Lusskin, Yannick Mahlich, Yanran Wang, Zishuo Zeng, Yana Bromberg

**Affiliations:** 1 Department of Biochemistry and Microbiology, Rutgers University, 76 Lipman Dr, New Brunswick, NJ 08873 USA; 2 Computational Biology & Bioinformatics - i12 Informatics, Technical University of Munich (TUM), Boltzmannstrasse 3, 85748 Garching/Munich, Germany; 3 TUM Graduate School, Center of Doctoral Studies in Informatics and its Applications (CeDoSIA), Technische Universität München, 85748 Garching/Munich, Germany; 4 Institute for Advanced Study, Technische Universität München, Lichtenbergstrasse 2 a, 85748 Garching, Germany

**Keywords:** Microbiome, Function analysis, Machine learning, mi-faser, MetaSUB

## Abstract

**Background:**

Accumulating evidence suggests that the human microbiome impacts individual and public health. City subway systems are human-dense environments, where passengers often exchange microbes. The MetaSUB project participants collected samples from subway surfaces in different cities and performed metagenomic sequencing. Previous studies focused on taxonomic composition of these microbiomes and no explicit functional analysis had been done till now.

**Results:**

As a part of the 2018 CAMDA challenge, we functionally profiled the available ~ 400 subway metagenomes and built predictor for city origin. In cross-validation, our model reached 81% accuracy when only the top-ranked city assignment was considered and 95% accuracy if the second city was taken into account as well. Notably, this performance was only achievable if the similarity of distribution of cities in the training and testing sets was similar. To assure that our methods are applicable without such biased assumptions we balanced our training data to account for all represented cities equally well. After balancing, the performance of our method was slightly lower (76/94%, respectively, for one or two top ranked cities), but still consistently high. Here we attained an added benefit of independence of training set city representation. In testing, our unbalanced model thus reached (an over-estimated) performance of 90/97%, while our balanced model was at a more reliable 63/90% accuracy. While, by definition of our model, we were not able to predict the microbiome origins previously unseen, our balanced model correctly judged them to be NOT-from-training-cities over 80% of the time.

Our function-based outlook on microbiomes also allowed us to note similarities between both regionally close and far-away cities. Curiously, we identified the depletion in mycobacterial functions as a signature of cities in New Zealand, while photosynthesis related functions fingerprinted New York, Porto and Tokyo.

**Conclusions:**

We demonstrated the power of our high-speed function annotation method, *mi-faser,* by analysing ~ 400 shotgun metagenomes in 2 days, with the results recapitulating functional signals of different city subway microbiomes. We also showed the importance of balanced data in avoiding over-estimated performance. Our results revealed similarities between both geographically close (Ofa and Ilorin) and distant (Boston and Porto, Lisbon and New York) city subway microbiomes. The photosynthesis related functional signatures of NYC were previously unseen in taxonomy studies, highlighting the strength of functional analysis.

## Background

The human microbiome, i.e. the microbial communities inhabiting various sites on and in the human body, is increasingly recognised as a critical component of human health [1]. Accumulating evidence associates the gastrointestinal (GI) microbiome with a wide range of multifactorial diseases, ranging from metabolic and immunological (e.g. diabetes [2, 3], Crohn’s Disease [4, 5]) to psychiatric (e.g. autism [6]) disorders. Skin microbiome has also gained increasing interest due to its association with various diseases [7–9]. City subway systems are human-dense environments, where interactions between passengers and the subway surfaces (i.e. handles, seats, walls and doors) provide fertile ground for microbe exchange. Notably, overall environmental factors, e.g. temperature and humidity, vary across different cities, contributing to the prosperity of different types of microbiomes in different cities. It is thus interesting from both ecological and public health perspective to study these differences. The MetaSUB project [10] profiles subway surface microbiomes from cities across the world via metagenomic sequencing. To date, a few studies have described, either via marker genes, e.g. 16S rRNA, or via genome-assembly, the microbiome taxonomic compositions [11–15]. However, to the best of our knowledge, no functional analysis has been attempted so far.

We recently created *mi-faser* [5], a computational method for super-fast (minutes-per-microbiome) and accurate (90% precision) mapping of sequencing reads to molecular functions of the corresponding genes. Our algorithmic advances are augmented by a manually curated reference database [5] of gene/protein enzymatic functionality. For the purposes of the 2018 CAMDA (Critical Assessment of Massive Data Analysis) challenge, we used *mi-faser* to functionally profile 392 MetaSUB metagenome datasets -- 310 samples from eight cities provided as the training set and 82 samples in need of evaluation/prediction, including eight training city and new city samples. We identified microbial functional signatures for each training city and built SVM (support vector machine) models to predict microbiome cities of origin.

Note that the training and test (evaluation) sets contain similar fractions of microbiomes for each of the eight cities. We demonstrated that balancing training data improves the performance of cities represented by fewer samples, i.e. avoids over-estimated performance. Notably, our balanced model made correct city assignments over 90% of the time (top two ranked cities), and correctly identified over 80% of the samples NOT from the training cities.

Our function-based outlook on microbiomes also allowed us to note similarities between both regionally close and far-away cities. We identified the depletion in mycobacterial functions as a signature of cities in New Zealand. We also found that the “concrete jungle”, i.e. New York City, subway microbiomes, as well as those from Porto and Tokyo, are best described by photosynthetic activity – a finding not seen via taxonomy studies.

## Methods

### Datasets and functional annotation

We obtained from the CAMDA (Critical Assessment of Massive Data Analysis) servers four MetaSub metagenome datasets: 1) *known set*, containing 310 metagenomes from AKL (Auckland), HAM (Hamilton), NYC (New York City), OFA (Ofa), PXO (Porto), SAC (Sacramento), SCL (Santiago) and TOK (Tokyo) subway systems; 2) *known-unknown* set, containing 30 samples from cities in the *known* set (later revealed to be 10 NYC, 10 PXO, 5 SCL, and 5 OFA); 3) *unknown set*, containing 36 samples from three new cities (later revealed to be 12 Ilorin, 12 Lisbon, and 12 Boston); and 4) *mix set*, containing 16 samples without further information (later revealed to be 3 from Boston, 5 from Bogota, 4 from Lisbon, and 4 from Ilorin).

All metagenomes were submitted to *mi-faser* [5] for quality control (Trim Glore [16], a wrapper tool around Cutadapt [17] and FastQC [18]) and function annotation. The resulting EC (Enzyme Commission [19]) number-based functional profiles produced by mi-faser were normalized by dividing the numbers of annotated reads per function by the total number of reads in sample. The maximum number of ECs that *mi-faser* can annotate is 1257 and the actual number of ECs annotated is microbiome dependent. For all MetaSUB samples in our set we used the union of all ECs as a vector of functions of each sample, replacing missing ECs by 0 s.

We additionally created two random sets: (1) a set of 1000 artificial metagenomes – to generate each sample in this true *random set*, we randomly selected ten samples from each city in the *known set* and, for each EC, picked an abundance value from these 80 samples at random; and (2) *random-label* set – the samples from the *known set* assigned randomly shuffled city labels (1000 times, resulting in 1000 random-label samples). Finally, we added one more set to our evaluation as negative control – an unrelated *SAND set* – the metagenomes collected from the beach sands in the Pensacola, Florida affected by the BP-oil-spill [20].

### Data modelling

#### 1) building predictors for each city using full functional (EC) profiles

For each city in the *known set*, we trained an SVM (support vector machine; e1071 R package [21]) model on the functional profiles of all samples in leave-one-out fashion to avoid overfitting. That is, 310 *raw-full* SVM models were built for each city, with one iteratively-selected sample removed from the *known set* prior to training. Note that we chose SVMs to model our data as, in our experience, they are better fitted to the task of dealing with sparse inputs; i.e. for each sample, many of the functions could be non-existent (while they do exist in other samples), thus their abundance were set to zero. Each SVM used 1252 features (ECs) to predict whether a given sample is from this city (positive) or any of the other cities (negative). The performance of each city predictor was evaluated by computing the AUC (area under curve; R pROC package [22]) under the ROC (receiver operating characteristic; true positive vs. false positive rate) and PR (precision vs. recall) curves (Eq. 1, 2, 3).

1 $$ false\ positive\ rate=\frac{False\ Positive}{True\ Negative+ False\ Positive} $$

2 $$ true\ positive\ rate= recall=\frac{True\ Positive}{True\ Positive+ False\ Negative} $$

3 $$ precision=\frac{True\ Positive}{True\ Positive+ False\ Positive} $$

#### 2) standardizing city predictor scores for final city assignment

We built a single SVM model for every city in the training set as described above but using the complete set of samples. Thus, each sample in our training data had been assigned a prediction score by each of the eight city predictors. For a given sample, these prediction scores were standardized individually for each city to the corresponding city range of scores of all other samples. The highest score was used for final city assignment. Note that this same (training) range of scores, as well as the rest of the standardization and city assignment procedure was used for all other samples in our study.

#### 3) identify city functional signatures

We further used the *dkm* feature selection algorithm [23] (CORElearn R package [24]) to select the top 20 signature ECs for each city. Note that this number of features was determined empirically by testing performance on sets of increasing numbers of ECs (5, 10, 20, 40; data not shown). We further trained *raw-select* SVMs to recognize individual cities as described above, using only the signature ECs selected in each iteration. Note that multiple top-20 EC sets were produced for each city cross-validation iteration; the 20 ECs most commonly selected in all iterations then became city functional signatures. The final city assignment was performed as described above by choosing the highest city score.

#### 4) remove data bias in the training set

As machine learning models benefit from data sets balanced for class representation [25] we resampled the *known set*, to produce equal numbers of positive and negative samples. Specifically, to avoid bias towards predominant cities (i.e. NYC and Porto), we resampled, with replacement, each of the city sets to 150 samples. We then resampled both negative and positive classes to produce 5000 samples each. We performed the same feature selection procedure and trained *balance-select* SVMs as described above, on the balanced data using the selected 20 ECs.

#### 4) build and evaluate final predictor model

Finally, we built a single *final* model for each city using the same procedure as for a single run of cross-validation of *balance-select* SVM model (feature selection, followed by SVM training on selected 20 ECs), but without leaving samples out. For all predictive evaluations reported here we used this model. We applied the *final* model to the *known set* of samples used in its development to obtain a measure of maximum performance that can be expected. We also applied it to the *random label* set to approximate the random baseline performance. We further compared the model predictions for the true *random set, SAND, known-unknown, unknown*, and *mix sets*. The features of the testing sets were standardized according to the training set features. For comparison purposes, we also built a *final-unbalanced* model for each city using the same procedure as for a single run of cross-validation of *raw-select* SVM (feature selection, followed by SVM training on selected 20 ECs), but without leaving samples out. We applied *final-unbalanced* SVM to the *known-unknown set*. Note that we didn’t correct for multiple hypothesis when we performed t-test identify enrichment and depletion of the EC signatures, as we didn’t use t-test to select these ECs from the entire list.

## Results and discussion

### City predictors are able to recognize sample cities of origin

**Fig. 1:**
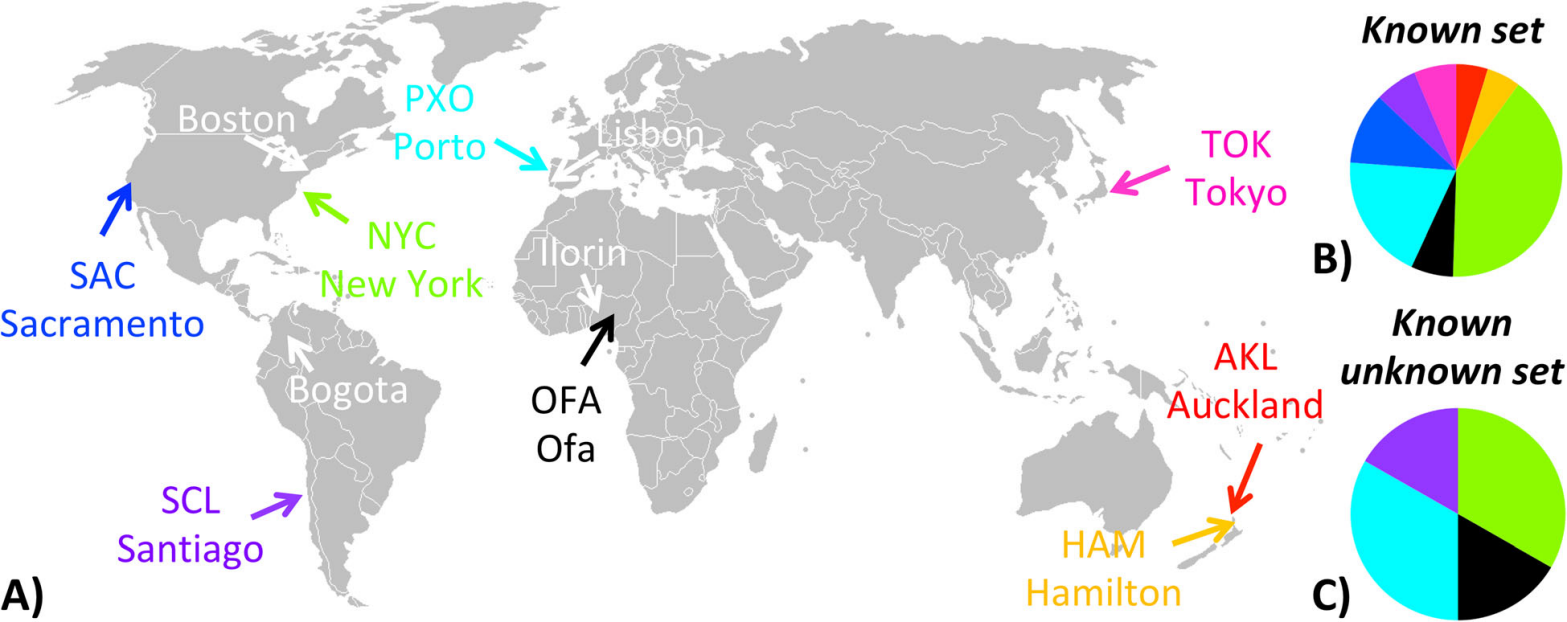
The city origins of the subway metagenomic samples. In **a**), the colored samples are from the *known* and *known-unknown sets*; the white samples are from the *unknown* and *mix sets*. Note that **b**) the *known set* and **c**) the *known-unknown* set are similarly dominated by NYC and Porto

We obtained EC (Enzyme Commission, [19]) number-based functional profiles of all the 392 shotgun metagenomic samples (all samples from the *known*, *known-unknown*, *unknown*, and *mix sets*) using *mi-faser* [5]. Note that using this tool the total computation took less than 2 days on a high-performance compute cluster with, on average, 500 cores available. *Known* and *known-unknown sets* contain samples from AKL (Auckland, New Zealand), HAM (Hamilton, New Zealand), NYC (New York City, USA), OFA (Ofa, Nigeria), PXO (Porto, Portugal), SAC (Sacramento, USA), SCL (Santiago, Chile) and TOK (Tokyo, Japan) subway systems, while *unknown set* and *mix set* samples were collected from Ilorin (Nigeria), Lisbon (Portugal), Boston (USA), and Bogota (Colombia; Fig. 1). Note that only the city origins of *known set* samples had been provided before the challenge.

**Fig. 2:**
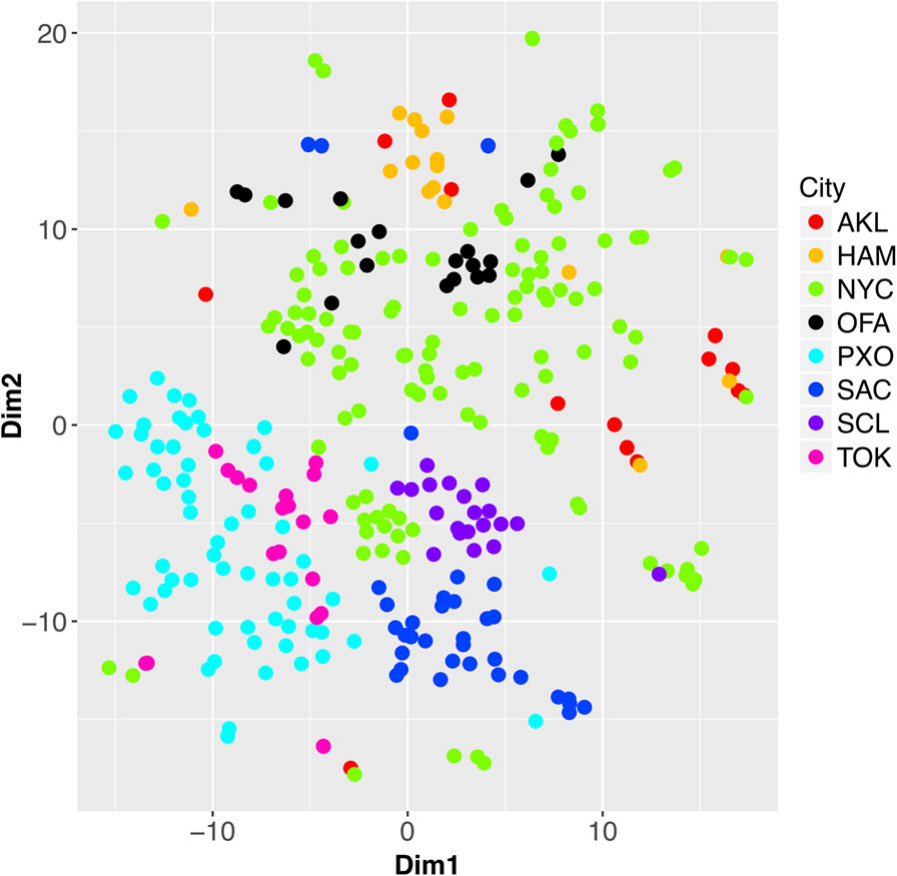
The functional profiles of the same city cluster together in the t-SNE plot [26]

In the *known set*, the functional profiles of the same city are significantly more similar to each other than to those of different cities (Fig. 2; p-val < 10e-3, Permanova test [27]). For each one of the eight cities in *known set*, we built a *raw-full* SVM (Support Vector Machine; full feature set of 1252 ECs; Methods) model [21, 28] to predict if a sample is from that city or not. We further selected the top 20 ECs (features; Methods) that best describe each city, and built, with only the selected ECs, *raw-select* SVMs for each city. In cross-validation, the AUCs (Area Under Curve) of the ROC (Receiver Operating Characteristic) curves were consistently high across the eight city predictors, for both *raw-full* (Additional file 1: Figure S1; AUC = 0.95 + 0.04) and *raw-select* (Additional file 2: Figure S2; AUC = 0.96 + 0.03) models. However, PR (precision vs. recall) curves varied more across cities for both *raw-full* (Additional file 1: Figure S1; AUC = 0.75 + 0.23) and *raw-select* (Additional file 2: Figure S2; AUC = 0.74 + 0.22) models. Note that this behaviour is not unexpected: while ROC curves measure how well both positive (“this city”) and negative (“not this city”) samples are classified, PR curves focus exclusively on the positive predictions. Hence PR measurements are more prone to fall victim to biased datasets; in our case, cities with few samples suffer (e.g. Auckland), while well-represented cities (e.g. New York City) are predicted well (Additional file 1: Figure S1 and Additional file 2: Figure S2).

Across our eight city predictors, the highest ranked city (highest normalized prediction score, Methods) was correct 78% (*raw-full*) and 81% (*raw-select*) of the time (Table 1). When we considered the top two city hits (instead of just one) performance was much higher, i.e. 90% (*raw-full*) and 95% (*raw-select*) (Table 1). The well-represented cities (e.g. New York City and Porto) were more likely to be correctly predicted by all models (Table 1). However, while the under-represented city samples were rarely highest ranked (e.g. Auckland *raw-select-SVM*, 33% recall), they were often second best (e.g. Auckland *raw-select-SVM*, 93% recall). Notably, when the under-represented city samples were correctly recognised as second ranked, the top hits were NYC or PXO over half the time (Table 1). This observation suggests that while our predictors could identify city-specific signals, they were affected by data imbalance.

**Table 1:** Assignment performance based on the eight city models

	*raw-full*						*raw-select*						*balance-select*					
	Top hit			Second hit			Top hit			Second hit			Top hit			Second hit		
	T^a^	F^a^	%	T^a^	F^a^	%	T^a^	F^a^	%	T^a^	F^a^	%	T^a^	F^a^	%	T^a^	F^a^	%
*AKL*	5	10	33	8	7	53	5	10	33	14	1	93	8	7	53	12	3	80
*HAM*	11	5	69	13	3	81	3	13	19	9	7	56	7	9	44	14	2	88
*NYC*	113	13	90	121	5	96	114	12	90	123	3	98	95	31	75	120	6	95
*OFA*	11	8	58	17	2	89	14	5	74	18	1	95	15	4	79	19	0	100
*PXO*	45	15	75	57	3	95	51	9	85	59	1	98	43	17	72	56	4	93
*SAC*	26	8	76	27	7	79	31	3	91	33	1	97	31	3	91	32	2	94
*SCL*	17	3	85	17	3	85	17	3	85	19	1	95	18	2	90	19	1	95
*TOK*	15	5	75	18	2	90	15	5	75	20	0	100	19	1	95	20	0	100
*All*	243	67	78	278	32	90	250	60	81	295	15	95	236	74	76	292	18	94

^a^Assignments are correct (T, true) if the sample provenance matches either of the two predicted cities, and incorrect (F, false) otherwise

### Data balancing helps with minor city identification

**Fig. 3:**
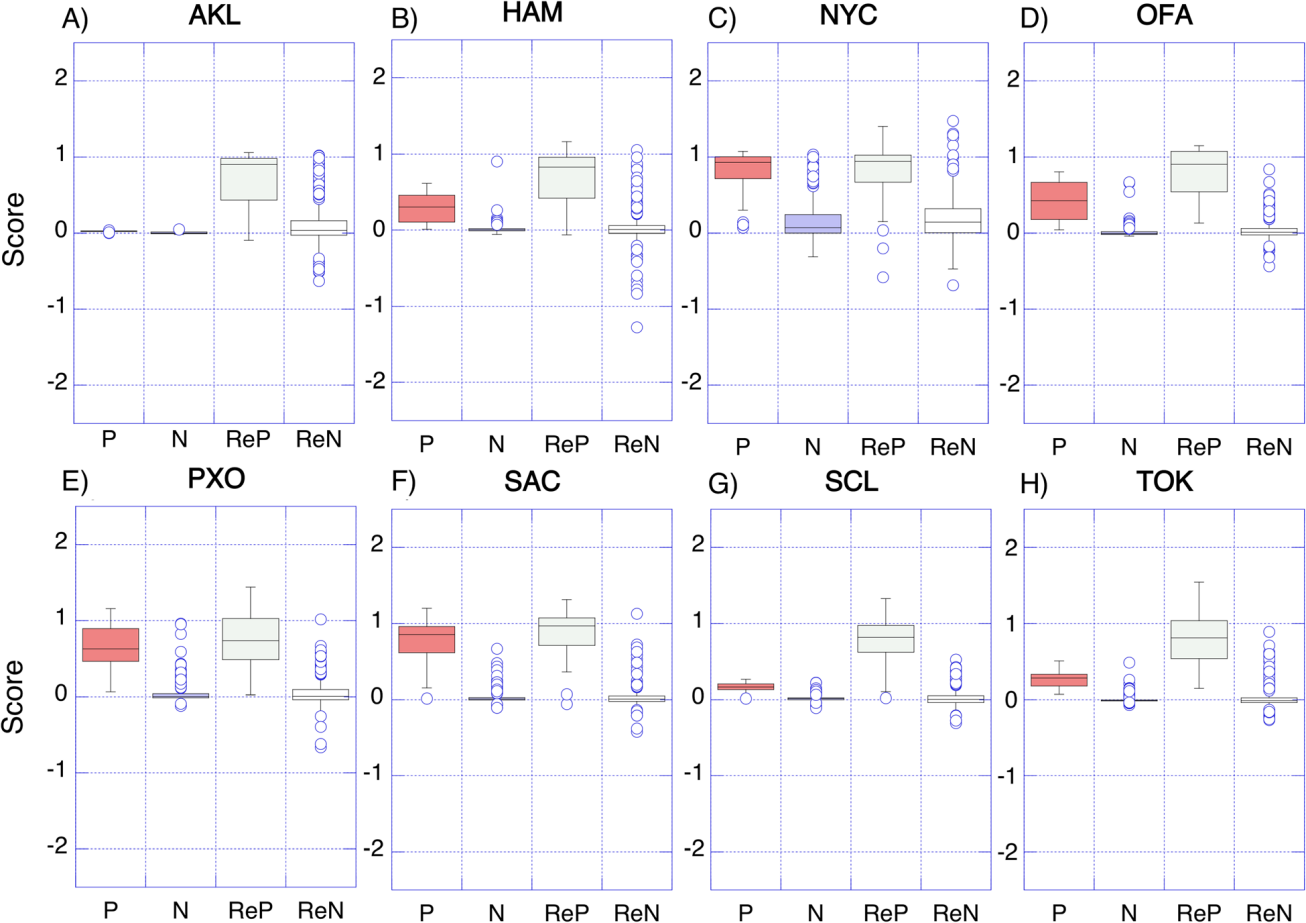
Distribution of prediction scores from the city predictors trained on the selected 20 ECs. **a** AKL (Auckland); **b** HAM (Hamilton); **c** NYC (New York City); **d** OFA (Ofa); **e** PXO (Porto); **f** SAC (Sacramento); **g** SCL (Santiago); **h** TOK (Tokyo). Positive (P) and negative (N) score distributions for raw-select models were less obvious to their resampled model (balance-select) versions (ReP and ReN)

In an effort to address the city imbalance problem described above, we resampled the *known set* to balance the representation of each city (Methods). While the difference in prediction scores between “this city” samples vs. “not this city” was already significant for even the unbalanced data models (*raw-select*; *p*-val < 10e-5, Kolmogorov–Smirnov test), data resampling (Methods; *balance-select*) drastically improved the differentiation. The distances between the average scores of positive and negative samples increased from 0.39 + 0.26 to 0.70 + 0.09, across the eight predictors (Methods; Fig. 3, P vs. N difference is less obvious than for ReP vs. ReN). As a result, the PR performance of the individual under-represented city predictors improved (e.g. for Auckland, the PR AUC went from 0.258 to 0.441 and for TOK: from 0.783 to 0.842; Additional file 2: Figure S2 and Additional file 3: Figure S3). However, the overall (final model) accuracy of city assignments dropped from 81 to 76% (Table 1), mostly due to the decreased recall of well-represented city samples (e.g. New York City went from 90 to 75% and Porto from 85 to 72%; Table 1). On the other hand, the under-represented city sample assignments improved (e.g. Auckland recall increased from 33 to 53%, Tokyo increased from 75 to 95%; Table 1).

In biased datasets, such as the *known set*, the assignment is often driven by the most common samples (here, best represented cities). This, however, changes performance for test sets with different city composition ratios. Since balancing training data improves performance regardless of class distributions [25], we built our *final* model using balanced data (Methods). This model predicted the *known-unknown set* samples with 63% recall (19 of 30 samples) when the top-ranked assignments were considered and 93% recall (28 of 30 samples) when the second highest hit was included (Table 2). Note that like the *known* training set, the *known-unknown* test set is similarly biased towards over-representing New York City and Porto (10 New York City and 10 Porto samples of 30 total; Fig. 1b and c). Thus, nine of the misclassified samples, which were from New York City and Porto, could have likely been better recovered by the *raw* models (Table 2). The fact that their balanced *final* model top rank assignments were Auckland and Tokyo (Table 2), however, suggests functional similarity of the microbiomes of Auckland vs. New York City and Tokyo vs. Porto. Note that the other 11 New York City and Porto samples in the set were correctly ranked highest (Table 2). To confirm our hypothesis, we trained the *final-unbalanced* model on raw data (Methods). This model correctly assigned all the previously misclassified New York City and Porto samples, strikingly, ranking Auckland and Tokyo second (Table 3) and reaching deceivingly high performance (90 and 97% recall for top and top-two hit assignments, respectively). In real life settings, i.e. without prior knowledge of city distributions, an unknown sample is equally likely to be from any city. Our results thus highlight the importance of balancing data for avoiding over-estimated performance.

**Table 2:**
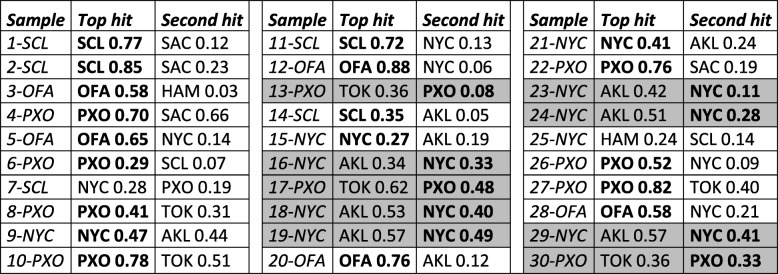
Final model scores for the *known-unknown* set

Boldface indicates correct hits. Shading indicates samples for which top hits are wrong and second hits are right

**Table 3:**
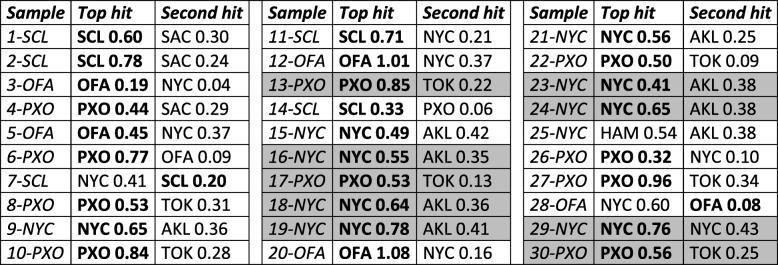
The top two city with highest normalized score (final-unbalanced) for the *known-unknown* set

Boldface indicates correct hits. Shading indicates samples for which top hits are wrong and second hits are right

### Predicting samples from previously unseen cities

**Fig. 4:**
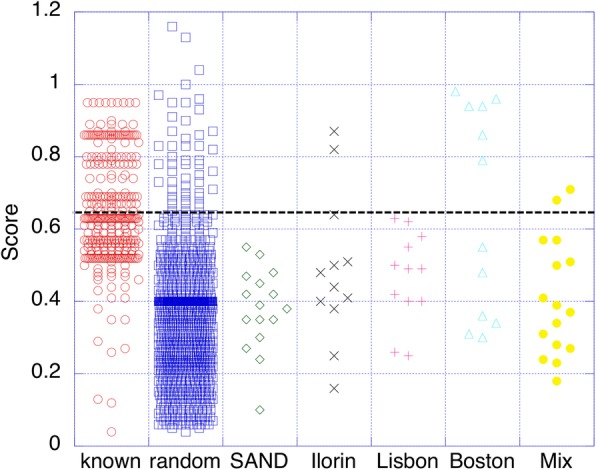
Distribution of top-match scores from *final-SVM*. The columns from the left are: known set, random set, SAND set, Ilorin samples from *unknown set*, Lisbon samples from *unknown set*, Boston samples from *unknown set* and *mix set*. The black dash line indicates 0.65, the cutoff below which the samples are likely to be random, i.e., the sample is not from any of the eight cities with which we trained our model

Our *final* model was built to recognize samples as coming from one of the eight training cities. Thus, using our top-hit approach, ANY metagenomic sample can be classified as coming from one of these cities – even if it doesn’t score high with the corresponding city model. To judge whether the sample had NOT come from any of the eight cities, we had to reconsider the samples where the top hit had a low score. We tested the *final* model city predictors on the *known set* vs. the *random set* (Methods). At the top-hit score = 0.65 there were fewer than 5% of the *random set* samples (Fig. 4). In other words, if a given unknown sample had a top-hit score > 0.65, we were more than 95% confident that it is from one of the eight known cities. As a validation experiment, we also note that none of the samples from the *SAND set*, an unrelated metagenome dataset (Methods), scored above this threshold.

In predicting the *unknown* and *mix sets*, both of which contain metagenomes from new cities (Fig. 1; Methods), our model correctly judged 81% (42 of 52) of the samples to be not from the eight known cities (Fig. 4). In the *unknown set*, two samples from Ilorin were assigned to Ofa, possibly due to the geographic adjacency (Figs. 1, 4). Strikingly, half of the Boston samples (6 of 12) were predicted to be from Porto (Fig. 4), which suggests strong similarity of the two cities’ subway microbiomes. On the other hand, despite of the regional proximity to Porto, none of the *unknown set* Lisbon samples scored above the threshold, while two *mix set* Lisbon samples were predicted to be from New York City (Fig. 4).

### Subway microbiome functional signatures reveal signals not seen by taxonomy studies

**Fig. 5:**
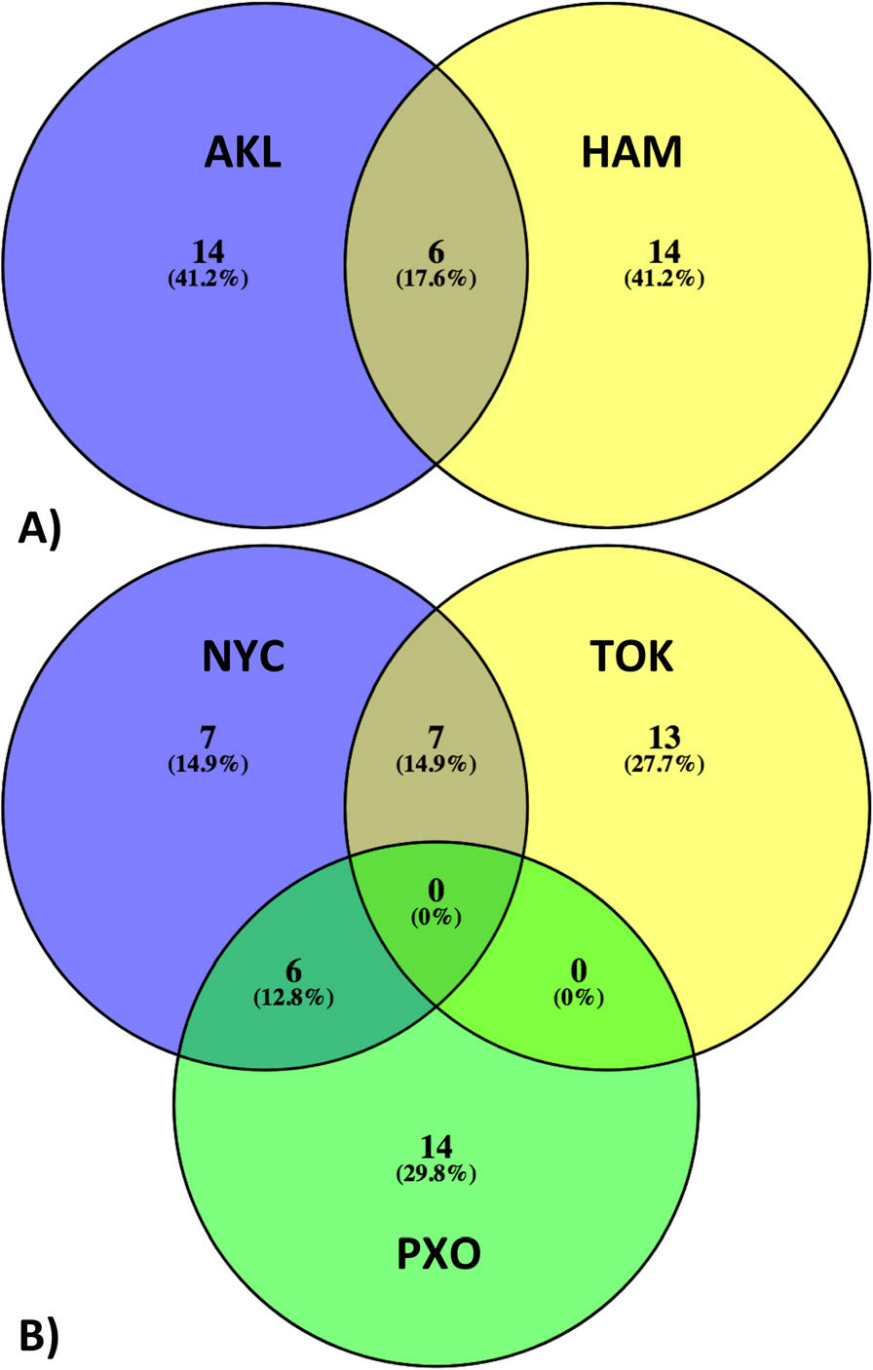
Venn diagrams [29] of city subway microbiome signature overlaps between **a**) AKL (Auckland) and HAM (Hamilton), and **b**) NYC (New York City), TOK (Tokyo) and PXO (Porto)

Here we showed that our 20 selected features/ECs are sufficient to differentiate city subway microbiomes. These ECs are, thus, the microbiome functional signatures of city subway systems (Additional file 4), where functional signatures shared by cities may indicate environmental similarity. For example, the two New Zealand cities, Auckland and Hamilton, share six of the 20 ECs (Fig. 5; Table 4). Two of the shared enzymes, EC 2.4.1.288 and EC 1.8.1.15 (Table 4) are associated with the *Mycobacterium* genus, a well-known source of human pathogens, e.g. *Mycobacterium tuberculosis* (MTB). Note that this association does not directly indicate the presence of MTB. The first of these enzymes is required for biosynthesis of arabinogalactan [30], a critical component of the unique mycobacterial cell wall structure essential for viability of MTB [31]. The second enzyme, which reduces mycothione to mycothiol, has been proposed as an MTB drug target [32]. Both enzymes were significantly depleted (p-val < 10e-5, t-test) in New Zealand cities as compared to the others, which is in line with low tuberculosis (TB) burden in New Zealand (0.23 incidences per 100,000 population, as compared to America (1.1 incidences per 100,000 population) and European (12 incidences per 100,000 population) regions) [33].

**Table 4:** The microbial functional signatures shared between AKL and HAM

EC	Annotation
1.8.1.15	mycothione reductase
1.8.7.1	assimilatory sulfite reductase (ferredoxin)
1.10.3.10	ubiquinol oxidase (H + -transporting)
1.2.2.3	formate dehydrogenase (cytochrome-c-553)
2.4.1.288	galactofuranosylgalactofuranosylrhamnosyl-N-acetylglucosaminyl-diphospho-decaprenol beta-1,5/1,6-galactofuranosyltransferase
5.4.3.5	D-ornithine 4,5-aminomutase

Another interesting observation is that New York City functional signatures overlap to a large extent with Porto and Tokyo (Fig. 5). Here, compared to other cities, NYC and Porto are depleted in these overlapping functions, while Tokyo is enriched in it (all enzyme *p*-vals<10e-5, t-test). Strikingly, most of these overlapping functions are associated with photosynthesis (Tables 5, 6). For example, New York City is depleted and Tokyo enriched in two enzymes, EC 1.3.7.5 and EC 1.3.5.5, which are involved in, respectively, porphyrin/chlorophyll metabolism and carotenoid biosynthesis [34]. Note that carotenoid pigments are not only able to harvest light energy on their own, but also protect chlorophyll from photodamage [35]. Notably, another four enzymes similarly enriched/depleted in Tokyo/NYC are associated with biochemical processes that are, mostly or exclusively, observed in cyanobacteria – a photosynthetic phylum (Table 5) [36–40]. On the other hand, function signatures similarly depleted in New York City and Porto include the photosystem II protein (EC 1.10.3.9), photosystem I iron-sulfur center (EC 1.97.1.12), enzymes (EC 3.2.1.31 and EC 6.6.1.1) involved in porphyrin and chlorophyll metabolism [34], and Ribulose bisphosphate carboxylase (RuBisCO; EC 4.1.1.39), the key enzyme in carbon fixation (Table 6).

**Table 5:** The microbial functional signatures shared between NYC and TOK

EC	Annotation
1.14.19.6	Delta(12)-fatty-acid desaturase
1.2.1.80^a^	Long-chain acyl-[acyl-carrier-protein] reductase
1.3.5.5^a^	15-cis-phytoene desaturase
1.3.7.5^a^	Phycocyanobilin:ferredoxin oxidoreductase
2.5.1.115^a^	Homogentisate phytyltransferase (HPT)
3.4.15.6^a^	Cyanophycinase
4.1.99.5^a^	Aldehyde decarbonylase (AD)

^a^Photosynthesis-related functions

**Table 6:** The microbial functional signatures shared between NYC and PXO

EC	Annotation
1.10.3.9^a^	Photosystem II protein
1.4.1.1	Alanine dehydrogenase
1.97.1.12^a^	Photosystem I iron-sulfur center
3.2.1.31^a^	Beta-glucuronidase (GUS)
4.1.1.39^a^	Ribulose bisphosphate carboxylase (RuBisCO)
6.6.1.1^a^	Magnesium-chelatase 38 kDa subunit

^a^Photosynthesis-related functions

For New York City, thus, our results thus suggest depletion in photosynthesis functionality (13 out of 20 signature functions). Note that *mi-faser* covers other bacterial photosynthesis-associated functions (EC 3.6.3.14 and EC 1.18.1.2, in KEGG pathway map00195) [34]. Though neither of these was selected as part of the New York City functional signature, both showed significantly lower abundance (*p*-val < 0.01, t-test), confirming our findings.

Interestingly, to the best of our knowledge and in contrast to our findings, none of the earlier MetaSUB taxonomic studies have reported detecting Cyanobacteria [11–15]. As these studies mostly addressed New York City samples, in which the photosynthetic functions are depleted, we fully expect this year’s taxonomy-focused MetaSUB studies to identify Cyanobacteria from the non-NYC samples. However, we are aware that they may not confirm our expectations, particularly for Tokyo, as no Cyanobacteria had been previously identified in the subway microbiome of another large modernized East Asian city, Hong Kong [41]. If indeed no Cyanobacteria are found, horizontal gene transfer may be to blame for the confusion. We have previously shown that, due in large part to horizontal gene transfer, bacterial taxonomy does not convey functional similarity [42] and that microbial functional diversification is driven by environmental factors [43]. It is also not hard to imagine that city subway environments, i.e. artificial light and high moisture and CO_2_ concentrations, select for photosynthetic activity.

Whether our results reflect taxonomy, or not, we suggest that functional analyses could reveal additional signals complementary to, if not more detailed and accurate than, taxonomic surveys.

## Conclusions

We used *mi-faser* to functionally profile 392 MetaSUB shotgun metagenomic samples. We demonstrated that 1) using test data with the same systematic bias as the training data leads to over-estimated performance and that 2) balancing biased training data improves prediction performance. Our predictor of microbiome city origins made correct city assignments > 90% of the time, and correctly judged samples to be NOT from training cities > 80% of the time. In addition, we found subway microbiome similarities between cities both geographically close (Ofa and Ilorin) and far (Boston and Porto, Lisbon and New York City). We identified mycobacterial functions as signatures for New Zealand cities, curiously implying persistence of public health risk in other cities. We also found that New York City, Porto, and Tokyo subway microbiomes are best described by both significant enrichment and depletion of photosynthetic functions, highlighting the strength of functional analysis.

## Supplementary information

**Additional file 1.** The ROC and PR curves of the eight city predictors in *raw-full* model.

**Additional file 2.** The ROC and PR curves of the eight city predictors in *raw-select* model.

**Additional file 3.** The ROC and PR curves of the eight city predictors in *balance-select* model.

**Additional file 4.** The microbiome functional signatures of city subway systems.

## Data Availability

The data are available from CAMDA website. The computational tools used in this study are referenced in the manuscript.

## Abbreviations

CAMDA: Critical assessment of massive data analysis
EC: Enzyme commission
PR curve: Precision vs. recall curve
ROC curve: Receiver operating characteristic curve
SVM: Support vector machine

## References

[1] Peterson J, Garges S, Giovanni M, McInnes P, Wang L, Schloss JA (2009). The NIH human microbiome project. Genome Res.

[2] Qin J, Li Y, Cai Z, Li S, Zhu J, Zhang F (2012). A metagenome-wide association study of gut microbiota in type 2 diabetes. Nature..

[3] Yassour M, Lim MY, Yun HS, Tickle TL, Sung J, Song Y-M (2016). Sub-clinical detection of gut microbial biomarkers of obesity and type 2 diabetes. Genome Medicine.

[4] Morgan XC, Tickle TL, Sokol H, Gevers D, Devaney KL, Ward DV (2012). Dysfunction of the intestinal microbiome in inflammatory bowel disease and treatment. Genome Biol.

[5] Zhu C, Miller M, Marpaka S, Vaysberg P, Rühlemann MC, Wu G (2018). Functional sequencing read annotation for high precision microbiome analysis. Nucleic Acids Res.

[6] Mulle JG, Sharp WG, Cubells JF (2013). The gut microbiome: a new frontier in autism research. Curr Psychiatry Rep.

[7] Casas C, Paul C, Lahfa M, Livideanu B, Lejeune O, Alvarez-Georges S (2012). Quantification of Demodex folliculorum by PCR in rosacea and its relationship to skin innate immune activation. Exp Dermatol.

[8] Clausen M-L, Agner T, Lilje B, Edslev SM, Johannesen TB, Andersen PS (2018). Association of Disease Severity with Skin Microbiome and Filaggrin Gene Mutations in adult atopic DermatitisSkin microbiome and gene mutations in adult atopic DermatitisSkin microbiome and gene mutations in adult atopic dermatitis. JAMA Dermatol.

[9] Fitz-Gibbon S, Tomida S, Chiu BH, Nguyen L, Du C, Liu M (2013). Propionibacterium acnes strain populations in the human skin microbiome associated with acne. J Invest Dermatol.

[10] Consortium MI (2016). The Metagenomics and Metadesign of the subways and urban biomes (MetaSUB) international Consortium inaugural meeting report. Microbiome..

[11] Zolfo M, Asnicar F, Manghi P, Pasolli E, Tett A, Segata N (2018). Profiling microbial strains in urban environments using metagenomic sequencing data. Biol Direct.

[12] Walker AR, Grimes TL, Datta S, Datta S (2018). Unraveling bacterial fingerprints of city subways from microbiome 16S gene profiles. Biol Direct.

[13] Qiao Y, Jia B, Hu Z, Sun C, Xiang Y, Wei C (2018). MetaBinG2: a fast and accurate metagenomic sequence classification system for samples with many unknown organisms. Biol Direct.

[14] Polewko-Klim A, Lesiński W, Mnich K, Piliszek R, Rudnicki WR (2018). Integration of multiple types of genetic markers for neuroblastoma may contribute to improved prediction of the overall survival. Biol Direct.

[15] Afshinnekoo E, Meydan C, Chowdhury S, Jaroudi D, Boyer C, Bernstein N (2015). Geospatial resolution of human and bacterial diversity with City-scale Metagenomics. Cell Syst.

[16] Krueger F (2012). Trim Galore.

[17] Martin M. Cutadapt removes adapter sequences from high-throughput sequencing reads. EMBnet.journal. 2011. 2011;17(1):3.

[18] Simon A (2010). FastQC: a quality control tool for high throughput sequence data.

[19] EC W. Enzyme nomenclature 1992: recommendations of the nomenclature Committee of the International Union of biochemistry and molecular biology on the nomenclature and classification of enzymes. San Diego: Academic press; 1992.

[20] Rodriguez-R LM, Overholt WA, Hagan C, Huettel M, Kostka JE, Konstantinidis KT (2015). Microbial community successional patterns in beach sands impacted by the Deepwater horizon oil spill. ISME J.

[21] Meyer D, Dimitriadou E, Hornik K, Weingessel A, Leisch F. e1071: Misc Functions of the Department of Statistics, Probability Theory Group (Formerly: E1071), TU Wien, 2015. R package version.1.6–7.

[22] Robin X, Turck N, Hainard A, Tiberti N, Lisacek F, Sanchez J-C (2011). pROC: an open-source package for R and S+ to analyze and compare ROC curves. BMC Bioinformatics.

[23] Dietterich T, Kearns M, Mansour Y. Applying the weak learning framework to understand and improve C4. 5: ICML. San Francisco: Morgan Kaufmann; 1996.

[24] Robnik M (2015). Package ‘CORElearn’.

[25] Wei Q, Dunbrack RL (2013). The role of balanced training and testing data sets for binary classifiers in bioinformatics. PLoS One.

[26] LJPvd M, Hinton GE (2008). Visualizing High-Dimensional Data Using t-SNE. J Mach Learn Res.

[27] Anderson MJ (2001). A new method for non-parametric multivariate analysis of variance. Austral Ecol.

[28] Cortes C, Vapnik V (1995). Support-vector networks. Mach Learn.

[29] Oliveros JC (2007). VENNY. An interactive tool for comparing lists with Venn Diagrams.

[30] Szczepina MG, Zheng RB, Completo GC, Lowary TL, Pinto BM (2009). STD-NMR studies suggest that two acceptor substrates for GlfT2, a bifunctional galactofuranosyltransferase required for the biosynthesis of Mycobacterium tuberculosis arabinogalactan, compete for the same binding site. Chembiochem.

[31] Alderwick LJ, Harrison J, Lloyd GS, Birch HL (2015). The mycobacterial Cell Wall--peptidoglycan and Arabinogalactan. Cold Spring Harbor Perspect Med.

[32] Rawat M, Av-Gay Y (2007). Mycothiol-dependent proteins in actinomycetes. FEMS Microbiol Rev.

[33] World Health Organization (2018). Global tuberculosis report 2018.

[34] Kanehisa M, Sato Y, Kawashima M, Furumichi M, Tanabe M (2016). KEGG as a reference resource for gene and protein annotation. Nucleic Acids Res.

[35] Armstrong GA, Hearst JE (1996). Carotenoids 2: genetics and molecular biology of carotenoid pigment biosynthesis. FASEB J.

[36] Klähn S, Baumgartner D, Pfreundt U, Voigt K, Schön V, Steglich C (2014). Alkane Biosynthesis Genes in Cyanobacteria and Their Transcriptional Organization. Front Bioeng Biotechnol.

[37] Savidge B, Weiss JD, Wong YH, Lassner MW, Mitsky TA, Shewmaker CK (2002). Isolation and characterization of homogentisate phytyltransferase genes from Synechocystis sp. PCC 6803 and Arabidopsis. Plant Physiol.

[38] Sattler SE, Cahoon EB, Coughlan SJ, DellaPenna D (2003). Characterization of Tocopherol Cyclases from Higher Plants and Cyanobacteria. Evolutionary Implications for Tocopherol Synthesis and Function. Plant Physiol.

[39] Richter R, Hejazi M, Kraft R, Ziegler K, Lockau W (1999). Cyanophycinase, a peptidase degrading the cyanobacterial reserve material multi-L-arginyl-poly-L-aspartic acid (cyanophycin): molecular cloning of the gene of Synechocystis sp. PCC 6803, expression in Escherichia coli, and biochemical characterization of the purified enzyme. Eur J Biochem.

[40] Paul B, Das D, Ellington B, Marsh EN (2013). Probing the mechanism of cyanobacterial aldehyde decarbonylase using a cyclopropyl aldehyde. J Am Chem Soc.

[41] Leung MHY, Wilkins D, Li EKT, Kong FKF, Lee PKH (2014). Indoor-air microbiome in an urban Subway network: diversity and dynamics. Appl Environ Microbiol.

[42] Zhu C, TO D, Vogel TM, Bromberg Y (2015). Functional basis of microorganism classification. PLoS Comput Biol.

[43] Zhu C, Mahlich Y, Miller M, Bromberg Y (2018). fusionDB: assessing microbial diversity and environmental preferences via functional similarity networks. Nucleic Acids Res.

