# Supplementary figures and images for: Fingerprinting cities: differentiating subway microbiome functionality
Source: Biol Direct. 2019 Oct 30;14:19. doi: 10.1186/s13062-019-0252-y (PMC6822482; doi:10.1186/s13062-019-0252-y)

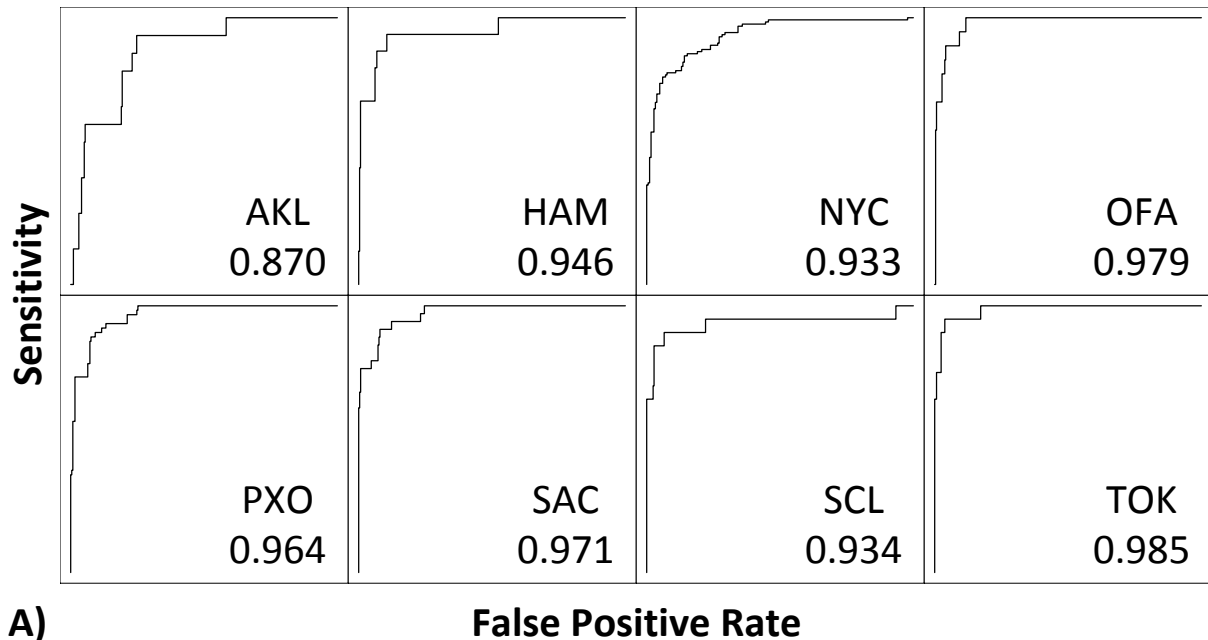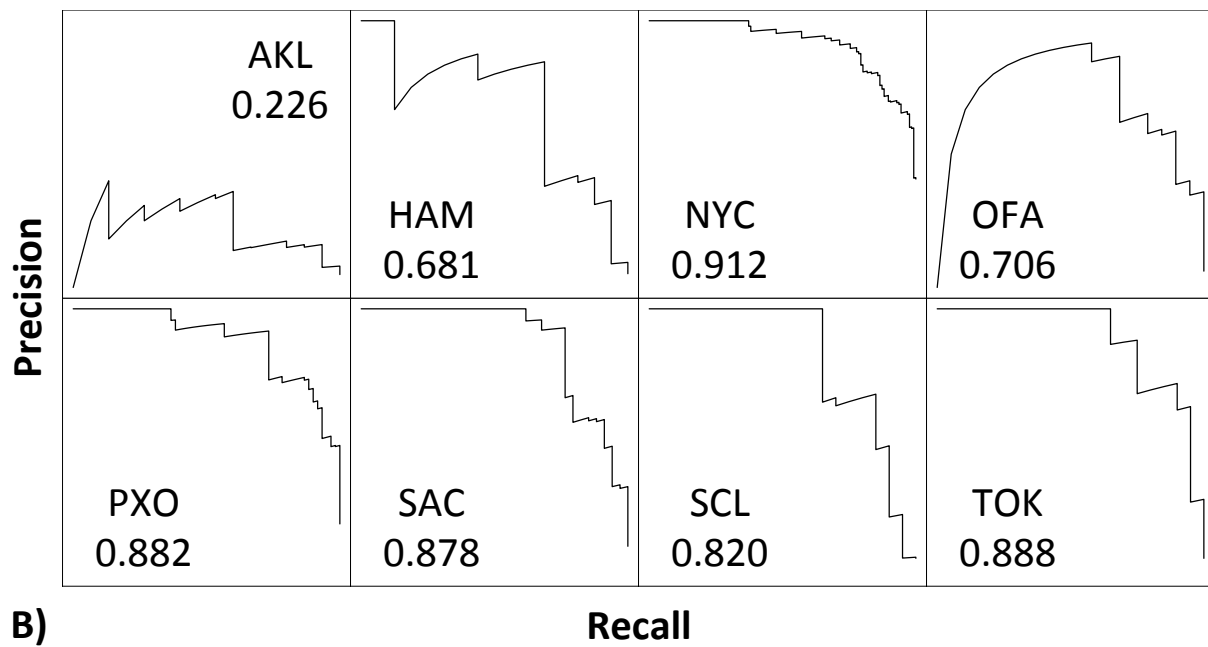

Supplement: Supplementary file 1 — Additional file 1. The ROC and PR curves of the eight city predictors in raw-full model. [file 13062_2019_252_MOESM1_ESM.pdf]

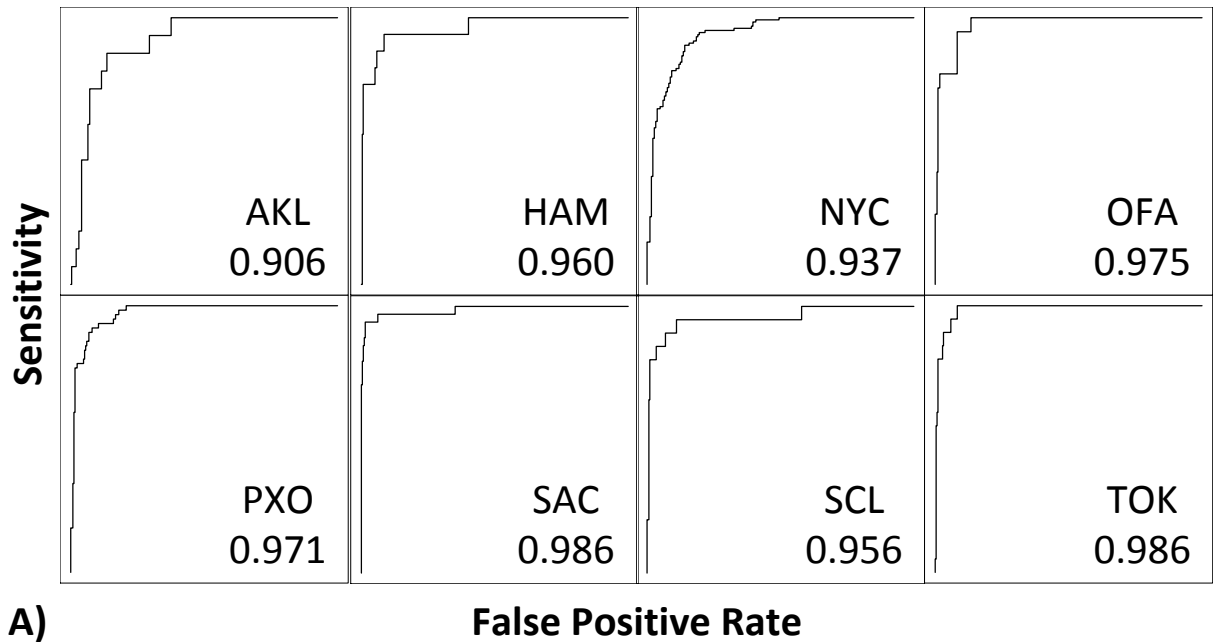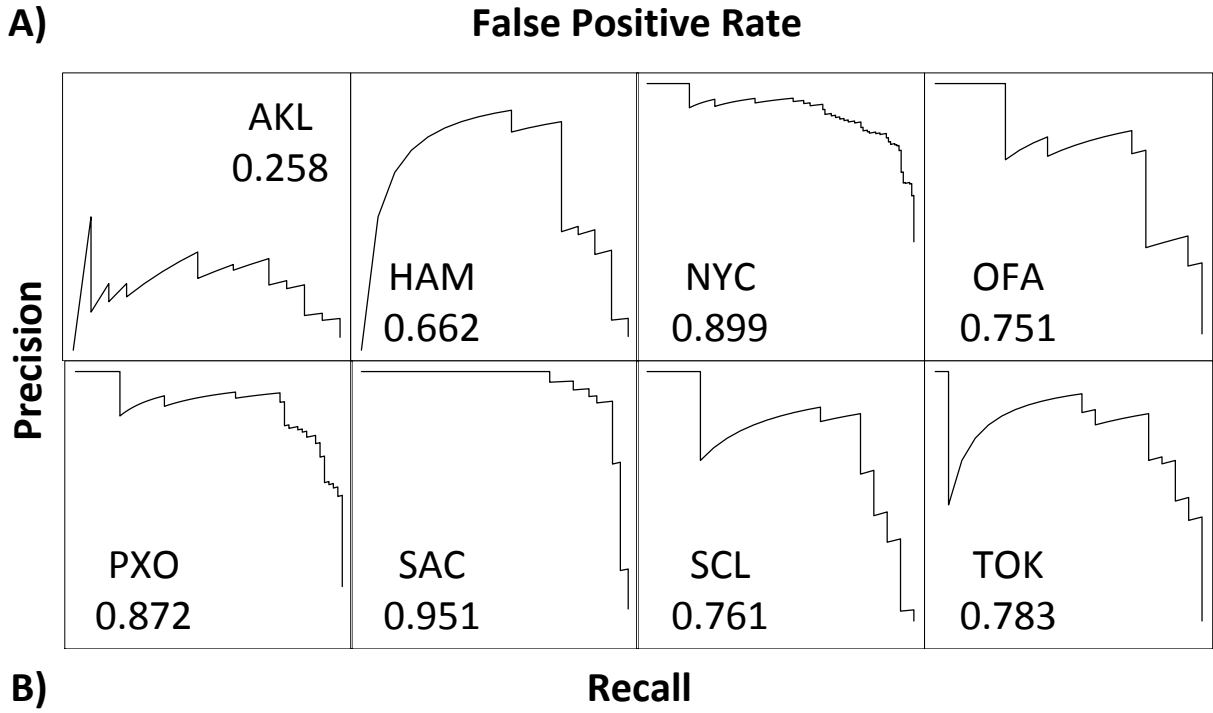

Supplement: Supplementary file 2 — Additional file 2. The ROC and PR curves of the eight city predictors in raw-select model. [file 13062_2019_252_MOESM2_ESM.pdf]

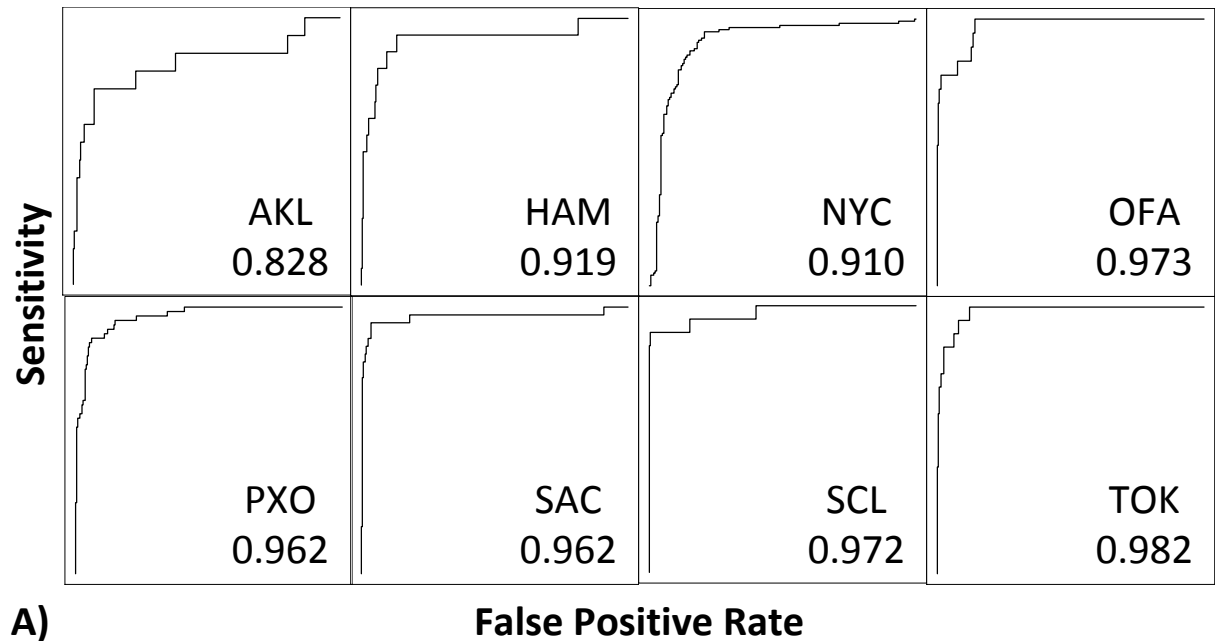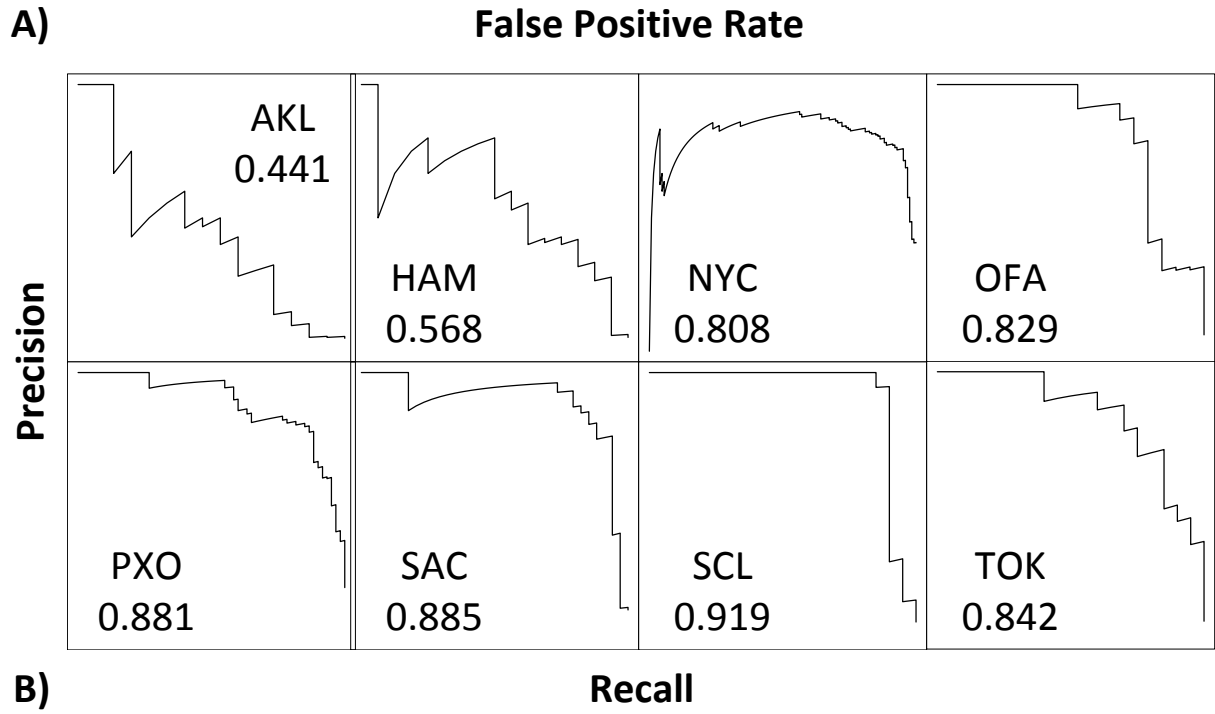

Supplement: Supplementary file 3 — Additional file 3. The ROC and PR curves of the eight city predictors in balance-select model. [file 13062_2019_252_MOESM3_ESM.pdf]
